# Vape store density and proximity to schools in Denpasar, Bali, Indonesia

**DOI:** 10.1136/tc-2023-058037

**Published:** 2023-08-04

**Authors:** Ni Komang Widiantari, Ni Made Dian Kurniasari, I Gusti Made Gde Surya Chandra Trapika, Putu Ayu Swandewi Astuti

**Affiliations:** 1 Bachelor of Public Health Program, Udayana University Faculty of Medicine, Denpasar, Indonesia; 2 Udayana Center for NCDs, Tobacco Control and Lung Health (Central), Udayana University, Bukit Jimbaran, Indonesia; 3 Department of Public Health and Preventive Medicine, Udayana University Faculty of Medicine, Denpasar, Indonesia; 4 Department Pharmacology and Therapy, Udayana University Faculty of Medicine, Denpasar, Indonesia

**Keywords:** electronic nicotine delivery devices, public policy, advocacy

## Abstract

**ABSTRACT:**

**Objective:**

Electronic cigarettes (e-cigarettes) use among youth in Indonesia is rising, and there is no regulation surrounding sale of e-cigarettes. This study aims to map the distribution and density of vape stores and their proximity to schools and cafes as well as assess selling of e-cigarettes to youth under 18 years in Denpasar, Bali.

**Methods:**

Using QGIS V.3.18.1 software, we conducted a geographic mapping of all vape stores followed by a survey of the retailers (n=107). Data were collected in April 2022. Several measures explored included retailers’ density based on the size and population of subdistricts, retailers’ proximity to school. Retailers were asked about selling to youth under 18 years, then its association with distance to schools and other variables were explored.

**Results:**

We mapped 122 vape stores across Denpasar city with a density of 1.56 per km^2^ of the occupied land for housing, 0.16 stores per 1000 total population and 1.06 stores per 1000 youth population. More than a quarter of the schools (28.3%) and the universities (25.6%) had at least one vape store in 250 m radius, while 97.2% of the stores were within 500 m of a café. Of the 107 vape store retailers interviewed, almost half (43.9%) reported selling vapes to youth under 18 years.

**Conclusions:**

Retail availability of e-cigarettes will contribute to the increasing use of this product, especially without a minimum legal sales age. The government should urgently prohibit selling to youth, regulate e-cigarette advertising, promotion and sponsorship and prohibit e-cigarette use where conventional smoking is prohibited.

**What is already known on this subject**

- There is increasing use of electronic cigarettes (e-cigarettes) among both adults and youth, and retail access to this product will influence its use.
- Conventional cigarettes retailers are widely available in neighbourhoods and around school in Indonesia; however, only one study in three provinces of Indonesia has mapped vape stores, finding more than half of selected vape stores were <500 m.
- A study in 10 cities of China, an upper middle-income country, found 5.7% of schools in urban and 1.9% in rural area had an e-cigarette retailer in a 100 m radius.

**What important gaps in knowledge exist on this topic**

- In low-income and middle-income settings, there is limited evidence on vape store density, proximity to school and youth meeting places, as well as retailers’ selling to youth under 18 years.

**What this study adds**

- This paper is among the first studies looking at the geographic distribution and density of vape stores and sales to youth in low-income and middle-income settings.
- This study found density of vape stores of 1.56 per km^2^ and 5.56% of school had a vape store in 100 m radius.
- The proportion of vape stores that reported selling to youth was as high as 43.9%.
- Evidence of widespread availability of e-cigarettes and sales to minors may encourage policy action to control it.

## Introduction

 Electronic cigarettes (e-cigarettes) use is becoming a global health concern.^1^ Between 2015 and 2018, the rate of e-cigarette use among adults ranged from 0.02% in India to 3.5% in Russia,^2^ while the youth prevalence ranged from 0.7% in Japan in 2017 to 23.4% in Poland in 2016.^3 4^ In Indonesia, adult e-cigarette increased 10-fold in 10 years from 0.3% in 2011 to 3.0% in 2021.^5^ The 2018 National Health Survey data showed that the e-cigarette use prevalence among people 10 years and above was 2.8%, while the prevalence at Kota Denpasar, the capital of Bali Province, was more than two times higher at 6.8%.^6^ The prevalence of e-cigarette use among youth aged 10–18 years in Bali was 20.8% in 2018,^6^ while a 2015 study found the prevalence of e-cigarette use among high school students aged 16–17 years in Denpasar was 20.5%.^7^

This increasing trend of e-cigarette use is also reflected in the sales revenue of e-cigarettes of Indonesian rupiah (IDR) 2.7 trillion (US$191.7 million) in 2018, which is projected to reach IDR 4.7 trillion (US$ 333.7 million) in 2023.^8^ In 2017, Indonesia had the second largest share of Instagram posts about vaping, based on the finding of a systematic random sampling of Instagram posts related to e-juice covering worldwide geographic area.^9^ While e-cigarettes may assist people to quit smoking, some raise concerns about e-cigarettes as a gateway to smoking especially among young people^10 11^ and also potency concerns of becoming dual users.^12^ Another concern is growing evidence of the health consequences of e-cigarette use.^13^

Meanwhile, in Indonesia, the majority of e-cigarettes are sold at the vape shops (64.7%) and online (35.3%).^14^ In Indonesia, there is no specific regulation on e-cigarettes except for an excise tax on e-cigarette liquid.^14^ Regulation on marketing and promotion is not yet adopted, allowing e-cigarette companies to employ different marketing channels and ease of access including among young people.^15^ There is a prohibition to sell cigarettes to young people under 18 years, which is in fact poorly enforced. However, there is no regulation in place for selling e-cigarettes to minors nor for retail proximity to school or other youth-populated venues.

The proximity and density of e-cigarette stores are associated with increasing use of e-cigarettes. For example, a US study documented one-third of the e-cigarette stores were located within two blocks from schools which may increase the likelihood of access among students to e-cigarettes,^16^ while in New Jersey, density of e-cigarettes retailers was positively associated with the use of e-cigarettes in the past month.^17^

A study in 10 cities across China, an upper middle-income country, found 5.7% of schools in urban and 1.9% in rural area had an e-cigarette retailer in a 100 m radius.^18^ A study that explore selected prominent vape stores in three provinces of Indonesia found more than half of the retailers reported their distance to a school was <500 m.^19^

With the growing trend of e-cigarette use including among young people, it is necessary to document its retail distribution to understand the availability and accessibility of e-cigarettes. The information can be considered by policy makers as a basis to develop regulation to reduce access and exposure to e-cigarettes environmental cues.

## Methods

### Study setting

This study was conducted in Denpasar, the capital city of Bali Province, one of the prominent tourist destinations in Indonesia. Data were collected in April 2022. Denpasar city is administratively divided into four subdistricts and the total population in 2021 was 726 599 people, while the youth population (aged 10–19 years) was 112 108 people. The total number of schools from primary to senior high schools was 379, and universities or equivalent was 43. The age range of students from primary to senior high school is between 6 and 18 years, while the age range of university students (undergraduate) is between 17 and 26 years.

### Data collection

In this paper, we reported the geographic information system (GIS) mapping and part of an audit survey of vape stores. The stores coordinates were collected by four teams of two trained undergraduate public health students. The teams explored all roads in the city except the small alleys which only fit motorbikes. The schools’ and the universities’ coordinates were geocoded based on the addresses retrieved from the reference data of the Ministry of Education and Culture following the steps of selecting level of education, then province, city/district and subdistrict (https://referensi.data.kemdikbud.go.id/). The number of population and size of the area were derived from Denpasar Bureau of Statistics. For the audit survey, all vape stores mapped during the GIS mapping was approached and inform consent was obtained from the shopkeepers prior to data collection.

### Measures

We calculated vape store density per 1000 total population and 1000 youth population (aged 10–19 years), and density based on the size of occupied land for housing in square kilometres (km^2^) overall and for each subdistrict. We also calculated the number of vape stores in 25, 100, 250 and 500 m radius from schools/universities, the number and proportion of schools/universities with at least one vape store in those radii and the straight line distance from schools/universities to the closest vape store. We linked to the audit survey data to measure the association between the retailer selling practice to youth under 18 years with the distance of the stores to schools and the location of the stores in relation to the main road, ease of access (the ability to find the shops including road condition and routes), visibility (whether the stores were visible from the main road or not) and availability of a café within 500 m of a vape store.

### Statistical analysis

Descriptive analysis was employed to get the number and percentage of vape stores in certain radius from schools and universities. We used χ^2^ test and Fisher’s exact test to measure the association between retailer’s selling to youth under 18 years and the radius of distance from schools and other variables. For this analysis, the distance was categorised into four groups: ≤100 m; 100.1–250 m; 250.1–500 m; >500 m. The geographic mapping was processed with QGIS V.3.18.1 and the statistical analysis was performed with STATA/IC V.15.1.

## Results

**Figure 1:**
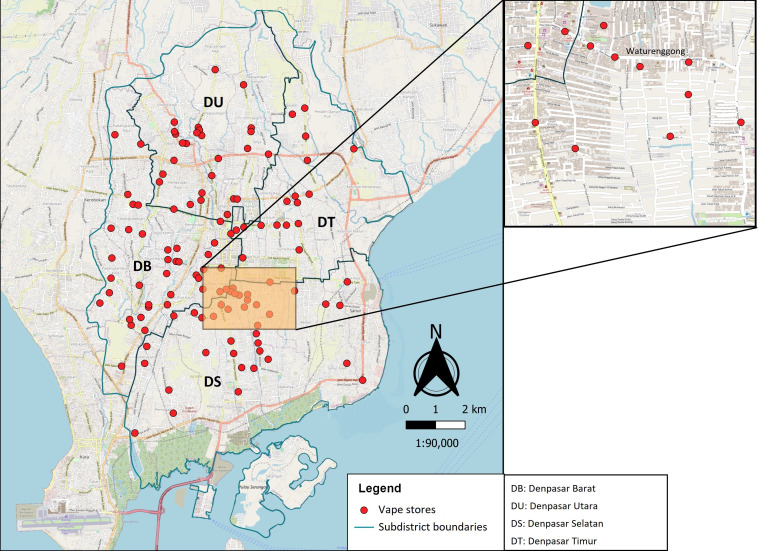
Spatial distribution of vape stores in Denpasar city in 2022 (inset: a street with high presence of vape stores).

We were able to find and map 122 vape stores across Denpasar city. Figure 1 shows that the vape stores are available in all subdistricts, with the highest number of 42 stores found in Denpasar Barat subdistrict accounting for 34.43% of all stores. In the inset of figure 1, the highlighted area depicts the more clustered vape stores. This was at Waturenggong Street which is part of Panjer urban village where rental housings for students and university students are prevalent.

The overall density of vape stores in Denpasar was 0.16 per 1000 total population, 1.06 per 1000 youth population and 1.56 per km^2^. The highest density based on these three aspects was found in Denpasar Barat subdistrict with 0.20 stores per 1000 total population, 1.30 stores per 1000 youth population and 2.24 stores per km^2^.

Of the 122 vape stores, 47.5% were located within 250 m radius from schools. Of the 378 schools, 5.6% have at least one vape store in 100 m radius and more than a quarter (28.3%) within 250 m radius, with a maximum of four stores (online supplemental table 1). Similarly, around a quarter (25.6%) of the universities in Denpasar have at least one vape store in 250 m radius (online supplemental table 1 and online supplemental figure 1).

For the audit survey, 107 vape stores were involved (response rate 88.7%), 10 shopkeepers declined to participate because they refuse to be interviewed, do not have time or were not allowed by their owner; and 5 stores were closed after two visits. Almost half (43.9%) of the vape shopkeepers admitted selling to youth under 18 years. We found no significant difference in selling to youth under 18 years based on proximity to schools, store location in regard to main road, ease of access, store visibility and its proximity to a café. However, we can see that half (50.0%) of the vape shopkeepers within 100 m radius from schools reported that they were selling vapes to youth below 18 years (online supplemental table 2).

## Discussion

The increasing trend of e-cigarettes use especially among young people in Indonesia reflects the high retail availability and accessibility of these products. The physical presence of e-cigarette stores is associated with the likelihood of higher access^20^ and environmental cues to e-cigarettes use including its advertising and promotion.^21^ Our mapping in Denpasar city found relatively high density of vape stores of 0.16 per 1000 total population and 1.06 per 1000 youth population, its close proximity to school and high rates of selling to youth under 18 years.

A previous study showed that e-cigarette stores are concentrated in the urban area with greater population density.^20^ Denpasar and Bali in general has become an area of prime target of new trends, for instance, the second IQOS store in Indonesia was established in a mall in Bali after the Indonesia’s capital, Jakarta. Although the density of vape stores is much lower than the density of conventional cigarette store mapped in Denpasar in 2019 at 4.6 per 1000 population,^22^ it is still concerning since about half of the stores are located near schools, on the big roads, with high visibility and accessibility.

Almost half of the vape stores in Denpasar were located within 250 m radius and almost three-quarters within 500 m from schools. This finding is much higher than in Central Texas, USA where 40% of the e-cigarette stores are located within 500 m radius from schools.^23^ Meanwhile, 5.6% of schools in Denpasar have at least one vape store in 100 m radius which is similar to the figure at urban area in China.

E-cigarette retailers density around school was positively associated with ever and past month use among high school student in New Jersey,^17^ signifying that the density of retailers can be a predictor of e-cigarette use. Our study suggests that e-cigarette businesses are adopting the conventional cigarette marketing strategies that effectively reach youth market including store location close to schools that increase the environmental cues to smoking initiation.^23 24^ This is reflected in the higher rate of e-cigarettes use among the youth compared with the adults.^6^

The proportion of vape shopkeepers in Denpasar who admitted to selling vapes to young people below 18 years is four times higher than observed sales to minors in four US states at 14.19%.^25^ The odds of selling to young people did not differ based on the distance from schools and the store location. Unfortunately, there is no regulation in Indonesia that prevent retailers from selling e-cigarettes to minors.

There are several measures that could be adopted by extending current regulations of conventional cigarettes, including prohibition of using e-cigarettes in smoke-free area; application of health warnings on product packaging and prohibition of advertising and promotions of e-cigarettes in all media channels.^26^ The government also has an option to control the permit of opening an e-cigarette store by adopting licensing policy, zoning policy and other retail control measures.

To our knowledge, this paper is among the first to report geographic mapping of e-cigarettes stores in low-income and middle-income countries, but is subject to some limitations. We did not include minimarkets and supermarkets that may also sell e-cigarettes, which may underestimate the retail availability of e-cigarettes. Therefore, future research should explore these types of retailers as well as online retailers. Regarding sale to minors, we asked “Are you selling e-cigarettes to everyone including youth and children?” This question may be subjective and the self-report may underestimate the rate of sales. However, there is no requirement to show identification card on purchasing e-cigarettes and the retailers may not be able to clearly identify which buyers are underage. We have used similar proxy question to assess cigarette retailer selling practice in a previous study.^22 27^ Further research may use direct observation of underage decoys to explore this practice.

In summary, we found vape stores are highly available and accessible to young people in areas close to youth meeting places such as schools. The Indonesian government must strengthen its tobacco control measures to include e-cigarettes and other alternative nicotine/tobacco products. The government can also take the initiative to restrict the permit of e-cigarettes stores especially in areas close to schools and prohibit selling to young people under 18 years.

## supplementary material

10.1136/tc-2023-058037 online supplemental table 1

10.1136/tc-2023-058037 online supplemental table 2

10.1136/tc-2023-058037 online supplemental figure 1
