# Supplementary material for: Vape store density and proximity to schools in Denpasar, Bali, Indonesia
Source: Tob Control. 2023 Aug 4;33(e2):e058037. doi: 10.1136/tc-2023-058037 (PMC11672059; doi:10.1136/tc-2023-058037)
Supplement: online supplemental table 1 [file tc-33-e2-s001.pdf]

Table S1. Distribution and proximity of e-cigarettes stores around schools and universities in Denpasar in 2022

**Radius**	**Institutions**		**Number of e-cigarette stores**
**Schools (n=378) ***	f	%	
Within 25 meters	1	0.3	0 – 1
Within100 meters	21	5.6	0 – 2
Within 250 meters	107	28.3	0 – 4
Within 500 meters	284	75.1	0 – 7
**Universities (n=43)**			
Within 25 meters	0	0	0 – 0
Within100 meters	4	9.3	0 – 1
Within 250 meters	11	25.6	0 – 3
Within 500 meters	32	74.4	0 – 6

*) one junior and senior high school located in the same site
