# Supplementary material for: Vape store density and proximity to schools in Denpasar, Bali, Indonesia
Source: Tob Control. 2023 Aug 4;33(e2):e058037. doi: 10.1136/tc-2023-058037 (PMC11672059; doi:10.1136/tc-2023-058037)
Supplement: online supplemental table 2 [file tc-33-e2-s002.pdf]

Table S2. Retailers reported selling of e-cigarettes to youth under 18 years old based on proximity to school in Denpasar in 2022

| Variables (n=107) | Selling to youth below 18 years old | | p |
|---|---|---|---|
| | Yes (f; %) | No (f; %) | |
| Distance from school* | | | |
| ≤100 meter | 5 (50.0) | 5 (50.0) | 0.89* |
| 100.1 – 250meter | 7 (36.8) | 12 (63.2) | |
| 250.1 – 500meter | 20 (46.5) | 23 (53.5) | |
| > 500meter | 15 (42.9) | 20 (57.1) | |
| Located on the main road | | | |
| Yes | 38 (44.2) | 48 (55.8) | 0.91 |
| No | 9 (42.9) | 12 (57.1) | |
| Easy to access | | | |
| Yes | 45 (43.3) | 59 (56.7) | 0.58* |
| No | 2 (66.7) | 1 (33.3) | |
| High visibility | | | |
| Yes | 43 (45.3) | 52 (54.7) | 0.43 |
| No | 4 (33.3) | 8 (66.7) | |
| Close to a café (500 meters) | | | |
| Yes | 45 (43.3) | 59 (56.7) | 0.58* |
| No | 2 (66.7) | 1 (33.3) | |

* Fisher Exact Test
