# Supplementary material for: Vape store density and proximity to schools in Denpasar, Bali, Indonesia
Source: Tob Control. 2023 Aug 4;33(e2):e058037. doi: 10.1136/tc-2023-058037 (PMC11672059; doi:10.1136/tc-2023-058037)
Supplement: online supplemental figure 1 [file tc-33-e2-s003.pdf]

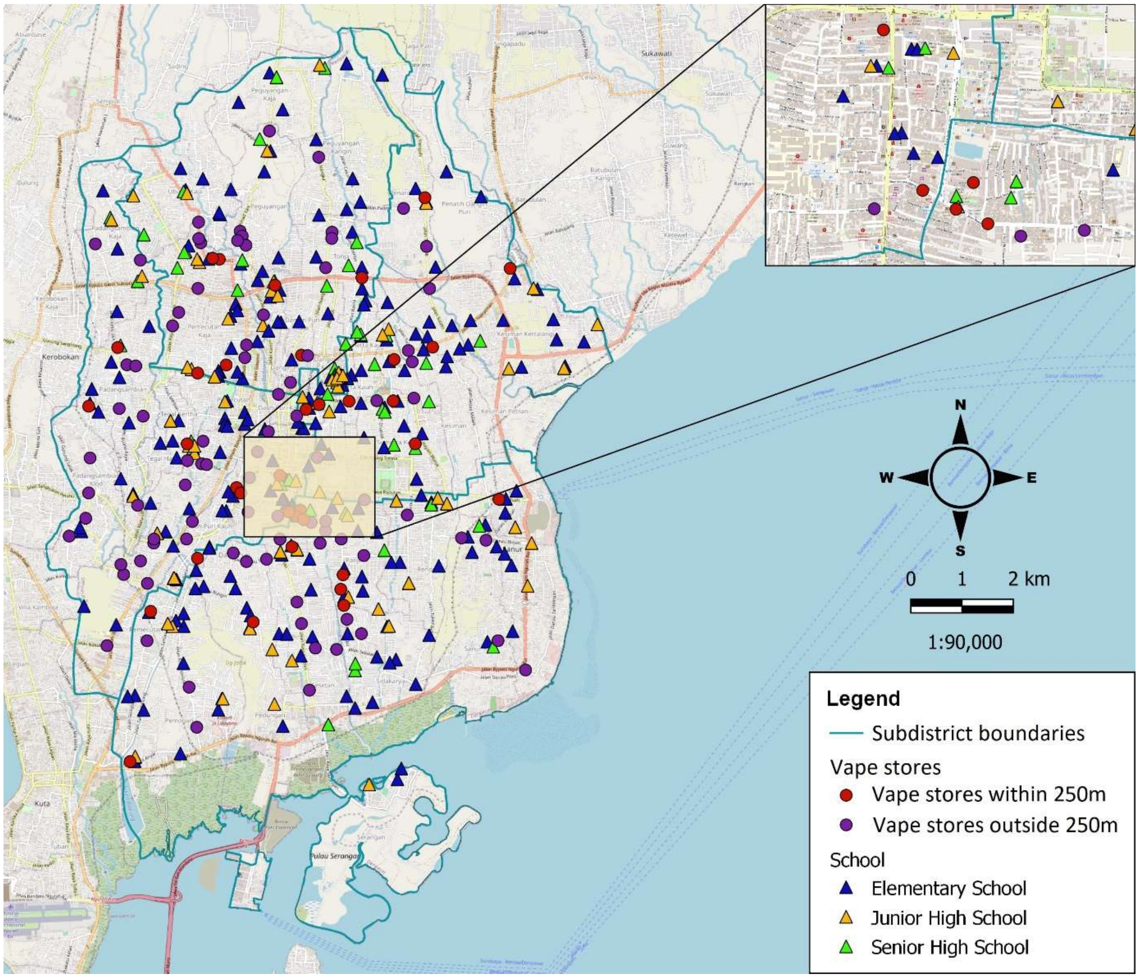

Figure S1. Spatial distribution of vape stores within and outside 250 m from schools (inset: a junior and senior high school with 4 vape stores in 250 m radius)
